# Growth Factor-Free Vascularization of Marine-Origin Collagen Sponges Using Cryopreserved Stromal Vascular Fractions from Human Adipose Tissue

**DOI:** 10.3390/md20100623

**Published:** 2022-09-30

**Authors:** Sara Freitas-Ribeiro, Gabriela S. Diogo, Catarina Oliveira, Albino Martins, Tiago H. Silva, Mariana Jarnalo, Ricardo Horta, Rui L. Reis, Rogério P. Pirraco

**Affiliations:** 1 3B’s Research Group, I3Bs—Research Institute on Biomaterials, Biodegradables and Biomimetics, University of Minho, Headquarters of the European Institute of Excellence on Tissue Engineering and Regenerative Medicine, Barco, 4805-017 Guimarães, Portugal; 2 ICVS/3B’s—PT Government Associate Laboratory, Braga, 4710-057 Guimarães, Portugal; 3 Department of Plastic and Reconstructive Surgery, and Burn Unity, Centro Hospitalar de São João, 4200-319 Porto, Portugal; 4 Faculty of Medicine, University of Porto, 4200-319 Porto, Portugal

**Keywords:** stromal vascular fraction, vascularization, blue shark skin collagen, 3D constructs

## Abstract

The successful integration of transplanted three-dimensional tissue engineering (TE) constructs depends greatly on their rapid vascularization. Therefore, it is essential to address this vascularization issue in the initial design of constructs for perfused tissues. Two of the most important variables in this regard are scaffold composition and cell sourcing. Collagens with marine origins overcome some issues associated with mammal-derived collagen while maintaining their advantages in terms of biocompatibility. Concurrently, the freshly isolated stromal vascular fraction (SVF) of adipose tissue has been proposed as an advantageous cell fraction for vascularization purposes due to its highly angiogenic properties, allowing extrinsic angiogenic growth factor-free vascularization strategies for TE applications. In this study, we aimed at understanding whether marine collagen 3D matrices could support cryopreserved human SVF in maintaining intrinsic angiogenic properties observed for fresh SVF. For this, cryopreserved human SVF was seeded on blue shark collagen sponges and cultured up to 7 days in a basal medium. The secretome profile of several angiogenesis-related factors was studied throughout culture times and correlated with the expression pattern of CD31 and CD146, which showed the formation of a prevascular network. Upon in ovo implantation, increased vessel recruitment was observed in prevascularized sponges when compared with sponges without SVF cells. Immunohistochemistry for CD31 demonstrated the improved integration of prevascularized sponges within chick chorioalantoic membrane (CAM) tissues, while in situ hybridization showed human cells lining blood vessels. These results demonstrate the potential of using cryopreserved SVF combined with marine collagen as a streamlined approach to improve the vascularization of TE constructs.

## 1. Introduction

Vascularization is a critical aspect of every tissue engineering (TE) approach for thick perfused tissues. A comprehensive network of capillaries is necessary to ensure, upon anastomosis with the host’s circulation, the proper delivery of nutrients and oxygen to all cells of the engineered tissue, avoiding necrotic events and promoting integration with the surrounding tissue. TE approaches for thick tissues not specifically addressing vascularization rely, in an initial phase after implantation, on passive diffusion, which is limited to ~150 µm [1]. Although the hypoxic environment created by the lack of oxygen potentiates the host vessel’s invasion towards the implanted construct, the rate of spontaneous vascular ingrowth is slow [2] and does not satisfy the metabolic needs of cells, leading to necrosis at the bulk of the graft. As a result, the successful use of TE constructs in the clinic is mainly limited to thin engineered constructs in which this rate of neovascularization from the host combined with diffusion is sufficient [3,4]. Therefore, most current strategies for engineering thick 3D constructs encompass a prevascularization step. Prevascularization uses endothelial cells (ECs) to form capillary-like networks before implantation, which will ideally anastomose with the circulation of the host tissue after implantation [5]. ECs are seeded alone or in combination with other supportive cell types and most frequently require supplementation with angiogenic growth factors [6,7]. However, the sourcing of ECs is an issue since macrovascular cells such as HUVECs are often used but are arguably not the most suited for capillary network formation [8]. Moreover, supplementation with extrinsic angiogenic growth factors fails to reproduce the growth factor kinetics involved in native vasculogenesis, which often leads to the formation of a non-mature network [9]. In this context, adipose tissue’s stromal vascular fraction (SVF) may be extremely important due to its intrinsic angiogenic potential [10]. This fraction can be isolated after the enzymatic digestion of adipose tissue obtained from liposuction or abdominoplasties, which otherwise would be discarded. Several cell populations encompass the SVF, namely endothelial and hematopoietic cells, mesenchymal and endothelial progenitors, pericytes, fibroblasts, and preadipocytes [10,11,12]. As previously reported, the pericytes and endothelial progenitors present in this fraction have the potential to spontaneously assemble in capillary-like networks in vitro without the need for angiogenic growth factor supplementation, both in 2D [13] and 3D settings [14]. However, freshly isolated SVF is usually used for this purpose, which may be disadvantageous for widespread clinical applications. The use of preserved SVF is therefore underexplored and warrants further investigation. Another important factor for the vascularization of 3D TE constructs is the biomaterial used. The use of natural origin polymers with intrinsic biocompatible properties has revealed a promising approach [15]. [15] Of these polymers, collagen is the most broadly used, as it is the major structural component of the native extracellular matrix (ECM) in living tissues and provides several cues for directing cellular behavior. Among the latter, adhesive motifs are included, which are powerful regulators of cell responses, such as cell spreading or stem cell differentiation [16]. Although collagen from mammalian sources is mostly used to produce TE constructs [17,18,19,20,21], regulatory and religious issues [22] have boosted the search for other collagen sources. Marine collagen is one such source. This type of collagen can be isolated from a number of marine species. In particular, the skin and bones of fish, sea urchin waste, jellyfish, and starfish have high collagen contents [15] with similar properties to mammalian collagen type I [23]. Due to by-catch, the blue shark is one of the most caught shark species [24], and its by-products are highly available and, thus, desirable for collagen extraction [25,26]. We have previously developed blue shark skin collagen sponges with interconnected micro-porous structures that promote not only human adipose stem cells adhesion but also ECM production and cell proliferation and infiltration within scaffolds, indicating a great potential for vascularization purposes [27].

Considering all of the above, blue shark collagen sponges were used as 3D matrices to explore the capacity of cryopreserved SVF to spontaneously yield a prevascular network in vitro in the absence of extrinsic angiogenic growth factors. This was achieved by assessing the spontaneous formation of capillary-like structures in vitro and by evaluating vessel recruitment, constructing the integration of the prevascular network with the host tissue after in ovo implantation using a chick chorioalantoic membrane (CAM) model.

## 2. Results

### 2.1. Generation of Prevascularized Collagen Sponges in an Extrinsic Angiogenic Growth Factor Free Manner

**Figure 1:**
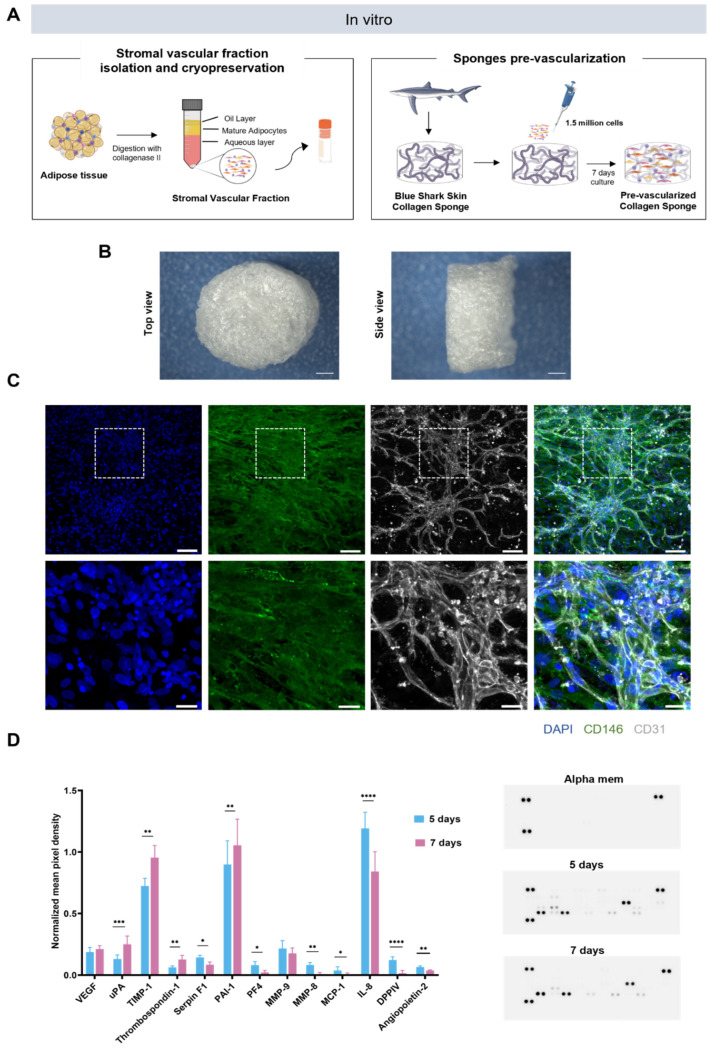
Generation of pre-vascularized collagen sponges. (**A**) In vitro experimental design. (**B**) Representative macroscopic images of shark skin collagen sponge’s macroporosity after freeze drying. Scale bar: 1000 µm. (**C**) Representative immunocytochemistry images of the network-like organization of SVF-derived CD31-expressing cells (white) interconnected with pericytes CD146-expressing cells (green), within collagen sponges after 7 days of culture in the absence of extrinsic angiogenic growth factors. Cell nuclei were counterstained with DAPI (blue). Scale bar: 75 μm (**top**) and 25 µm (**bottom**). (**D**) Angiogenic secretome profile of SVF cells seeded in collagen sponges at different culture periods. Conditioned media were collected at days 5 and 7 for dot blot analysis of angiogenesis-related factors. Protein expression profiles were measured using mean intensity and normalized to the reference spots. Data are presented as mean ± std dev and were analyzed using a paired *t*-test (* *p* < 0.0332, ** *p* < 0.0021, *** *p* < 0.0002, and **** *p* <0.0001).

We investigated the potential of cryopreserved SVF to create a prevascular network, in the absence of extrinsic angiogenic growth factors, after seeding in a blue shark collagen sponge (Figure 1A). Highly interconnected microporous sponges were produced by resorting to a cryogelation method, as previously described [27] (Figure 1B). SVF was isolated from human adipose subcutaneous tissue and cryopreserved in 10% DMSO in FBS for at least 7 days. SVF was then thawed and seeded on collagen sponges and cultured for 7 days without angiogenic growth factor supplementation, as previously described for fresh SVF [13] (Figure 1A). After that period, the expression pattern of endothelial marker CD31 and pericytes CD146 revealed the presence of endothelial cells together with pericytes organized in a complex and interconnected capillary-like network, confirming that SVF maintained its capacity to create a prevascular network without angiogenic growth factor supplementation even after cryopreservation (Figure 1C).

### 2.2. Profiling of Angiogenesis-Related Proteins in Prevascularized Collagen Sponges Secretome

Given the confirmation of prevascular network formation, we sought to understand if and how the secretome profile changed throughout the culture time. To achieve this, a multiplex analysis of secretome targeting angiogenesis-related proteins was performed on secretome samples collected after 5 and 7 days of culture (Figure 1D). The selection of these timepoints was based on previous studies from our lab [13]. The secretion of important angiogenic modulators such as VEGF and MMP-9 remained unchanged from one time point to the other. However, an increase in the secretion of several factors specifically involved in ECM remodeling (uPA, PAI-1, and TIMP-1) was verified from day 5 to 7, while macrophage-related factors decreased over culture time (IL-8, MCP-1). Interestingly, no expression of angiogenic proteins other than the ones detected for the first timepoint was found for the later timepoint.

### 2.3. In Ovo Evaluation of Angiogenic Potential

**Figure 2:**
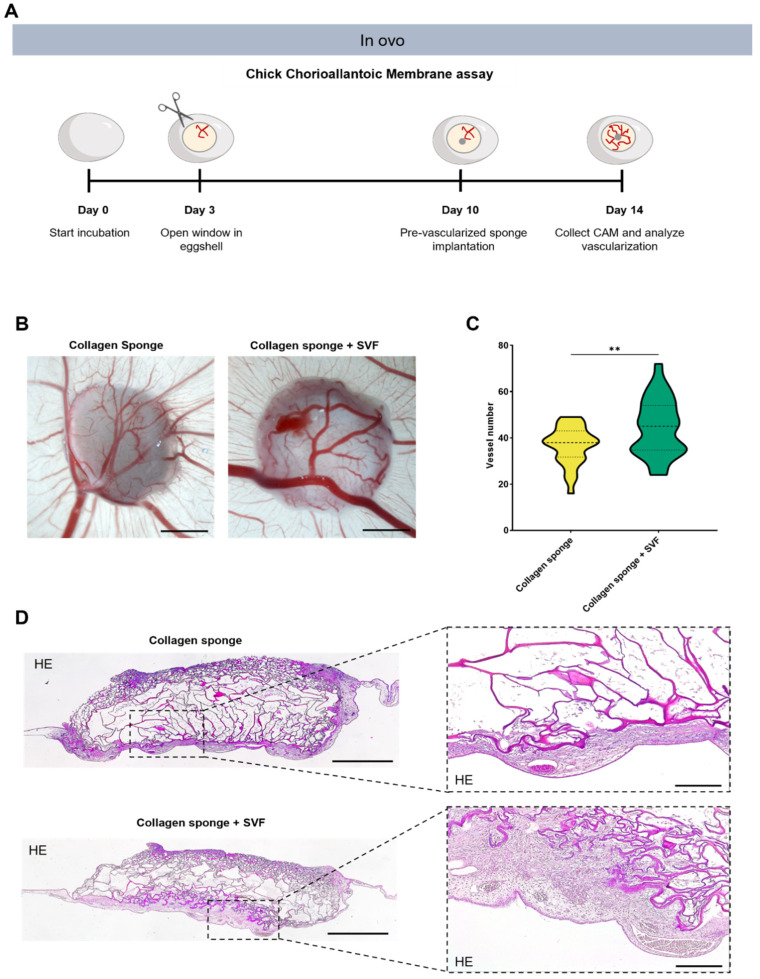
In ovo angiogenic potential upon implantation in Chick Chorioallantoic Membrane (CAM). (**A**) In vivo experimental design. (**B**) Representative micrographs of recruited vessels after 4 days of implantation of collagen sponges with and without SVF. Scale bar: 2000 µm. (**C**) Quantification of recruited vessels after 4 days of implantation of collagen sponge with and without SVF. Data are presented as violin plot illustrating the kernel density distribution frequency of recruited vessels and analyzed using an unpaired *t*-test (** *p* < 0.0021). (**D**) Representative micrographs of hematoxylin and eosin staining in collagen sponges with and without SVF. Scale bar: 500 μm (**left**) and 50 μm (**right**).

**Figure 3:**
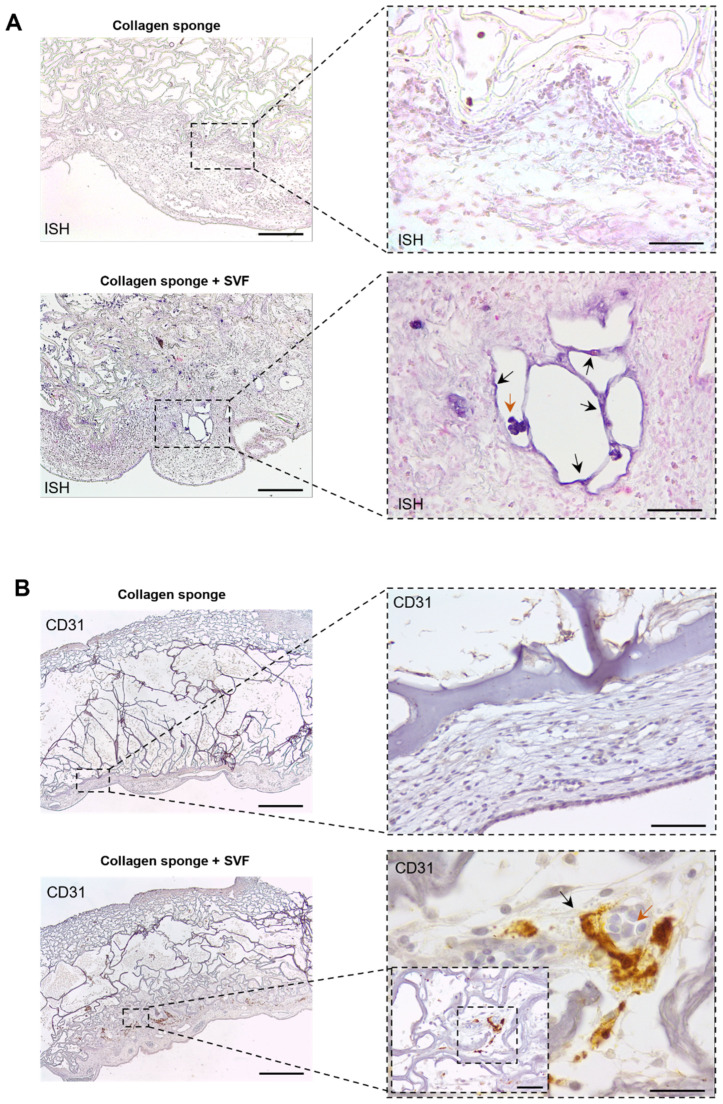
In ovo angiogenic potential upon implantation in Chick Chorioallantoic Membrane (CAM). (**A**) Representative images of the in situ hybridization performed with a DNA probe that stains human cellular nuclei (blue, arrows) in contrast with chicken nuclei (pink). The implanted cells infiltrated the host tissue and vasculature as highlighted by black arrows. Chicken erythrocytes identified by orange arrows. Scale bars: 200 μm (**left**) and 50 μm (**right**). (**B**) Representative immunohistochemistry images of the collagen sponge after 4 days of implantation showing human CD31-positive cells (brown). Human CD31 expression patterns demonstrated the integration of the pre-vascular network in the CAM, as highlighted by black arrows. Chicken erythrocytes identified by orange arrows. Scale bars: 500 μm (**left**) and 20 μm (**right**) and 50 µm (**inset right**).

Upon the in vitro confirmation of prevascular network formations, the in ovo angiogenic potential of the prevascularized collagen sponge was assessed by using a chick CAM assay (Figure 2A). After prevascularization, collagen sponges were implanted into the CAM of chicken eggs. A control group consisting of sponges without seeded cells was also implanted. For the evaluation and quantification of angiogenesis, the area around the implantation site was fixed, photographed, and finally excised and paraffin embedded. Results demonstrate host vessel recruitment in both the prevascularized and control sponges (Figure 2B). However, vessel quantification demonstrated a significantly higher number of recruited vessels for prevascularized sponges when comparing with sponges without SVF cells (Figure 2C), strongly suggesting a beneficial role of prevascularization with SVF in post-implantation vascularization. Concurrently, histological analysis after H&E staining clearly presented a higher CAM tissue ingrowth towards the bulk of prevascularized sponges in contrast with the control group with empty sponges where host tissue was very much limited to the outside of the sponge’s structure (Figure 2D). Importantly, no significant immune reaction was visible for both groups. Together with the higher number of recruited vessels, these results strongly suggest a positive effect of prevascularization with SVF upon the integration of implanted collagen sponges with the CAM tissue. In situ hybridization results show that human origin cells from the SVF persist in the CAM tissue after 4 days of implantation (Figure 3A) and, importantly, incorporate new vessels, suggesting a net contribution to the higher vessel density determined above. The contribution of implanted SVF cells to neo-vessel formation was further confirmed after immunohistochemistry for human CD31, which clearly demonstrates CD31-positive cells lining blood vessel walls in the interface between CAM tissues and collagen sponges (Figure 3B).

## 3. Discussion

The fast and efficient establishment of blood perfusion in engineered constructs after transplantation represents one of the major challenges for the incorporation of TE products into the clinical practice. Some early vascularization approaches focused on vascular ingrowth stimulation in tissue constructs by optimizing scaffolds’ material properties [28] or by incorporating growth factor delivery systems [29,30]. Both strategies proved to be inefficient, however, since vascular ingrowth is a slow process [2]. To overcome this, TERM strategies started to incorporate the in vitro creation of a prevascular network. ECs are seeded in scaffolds and supplemented with angiogenic growth factors that induce a vasculogenic-like process, ultimately forming a prevascular network [31]. However, the native vasculogenic process requires different growth factors produced by different cell types that interact with target receptors in a coordinated manner to ultimately yield a mature vascular network [9]. This is why early prevascularization strategies using only ECs, typically HUVECs, were often found to yield non-mature prevascular that is prone to regression after some period of time [32]. However, to recapitulate, the in vitro complexity of native vasculogenesis is a formidable challenge, both technically and in terms of costs due to the diverse cell types and different culture media and growth factors required. These facts urge the development of a streamlined and more cost-effective strategy to promote the vascularization of constructs.

In recent years, the SVF of adipose tissue became a focus of attention mainly due to its intrinsic angiogenic potential. However, studies demonstrating the angiogenic potential of SVF commonly use freshly isolated samples, which may limit its clinical applicability to specialized centers. The development of cell-banking strategies for SVF while maintaining its angiogenic potential would boost its clinical potential. The development of cell-banking strategies for SVF while maintaining its angiogenic potential would boost its clinical potential. Some studies describe the use of cryopreserved SVF for the production of vascularized adipose tissue, showing its ability to create capillary-like structures after preservation [33]. However, such studies use supplementation with angiogenic growth factors to induce capillary-like structure formation, which introduces a significant degree of complexity to the system. A previous study from our laboratory has shown that fresh SVF can spontaneously produce capillary-like structures as early as 5 days of culture, without the addition of angiogenic growth factors, representing a cost reduction and a more organic vasculogenic process since it is orchestrated by the cells themselves [13]. In this sense, we explored if cryopreserved SVF, in the absence of extrinsic angiogenic growth factors, retained this ability to spontaneously create a prevascular network in 3D conditions. SVF consists of a heterogeneous population of cells with an intrinsic capacity to secrete several angiogenic-associated growth factors, creating the perfect angiogenic microenvironment capable of promoting the formation of in vitro capillary-like networks in the absence of extrinsic angiogenic growth factors [13].

Vasculogenesis and angiogenesis are complex processes involving the coordinated action of several families of growth factors such as the vascular endothelial growth factor (VEGF), platelet-derived growth factor (PDGF), tissue inhibitor of metalloproteinases (TIMP), fibroblast growth factor (FGF), angiopoietin (ANG), interleukins (ILs), and matrix metalloproteinases (MMPs). All these players contribute not only ro capillary formation but, critically, to their stabilization and maturation [13]. In the 3D SVF cultures reported herein, we verified the protein expression of several angiogenic modulators over the culture time. Factors involved in ECM remodeling, namely urokinase (uPA), plasminogen activator inhibitor (PAI-1), and TIMP-1, increased from day 5 to day 7. Plasminogen activators such as (uPA) are key mediators of the ECM degradation process by converting inactive plasminogen to active plasmin, and it is in turn capable of degrading specific matrix constituents and of activating matrix-degrading metalloproteases such as MMP-9 [34]. Concurrently, it has been demonstrated that the increased production of uPA by microvascular ECs in response to angiogenic stimuli is accompanied by an increase in the production of the PAI-1 [35], which is in line with what we observed. This is most likely related with the need of a proteolytic balance that allows ECM degradation for cell migration but in a controlled fashion to keep the three-dimensional matrix intact, into which ECs form capillary-like networks [36]. The secretion of MMPs inhibitors is also influenced by this synergistic effect. Increased TIMP-1 expression is important for vessel stabilization by limiting matrix degradation and allowing matrix depositions that could explain its increase over culture times [37]. The decrease in IL-8 and MCP-1 expression also suggests that the angiogenic process is more directed to capillaries stabilization and maturation. In an initial phase of angiogenesis, MCP-1 recruits macrophages [38] that in turn secrete a variety of angiogenic-related factors such as thrombospondin-1 and IL-8 [39]. The latter is in fact essential for EC proliferation and migration [40,41], allowing capillary formation and elongation. The role of MCP-1 in angiogenesis promotion is not exclusively for macrophage recruitment. It up-regulates VEGF expression [42], increases vascular permeability [43], and is involved in pericytes recruitment [44]. The incorporation of pericytes initiates the stabilization and maturation of new vessels [45]. They are capable of modulating the ECM remodeling capacity of ECs by inducing the upregulation of PAI-1 in ECs and, in this way, limiting its migration and branching [46]. We verified the presence and incorporation of pericytes in the pre-vascular nerwork after 7 days, together with an increase over culture time of the levels of thrombospondin-1 and a decrease in levels of IL-8 and MCP-1 and stagnation in VEGF release. This strongly suggests a move towards the stage of capillary stabilization and maturation. This is further reinforced by decreased levels of ANG-2, involved in angiogenesis initiation [47]. The synergy between all cells present in the SVF and the secretion of all of these factors ultimately led to the formation of a prevascular network, as demonstrated by CD31’s expression pattern, and it is anchored by perivascular cells identified by the expression of CD146 and the lack of expression of CD31. Although the present study did not directly compare the angiogenic capacity of cryo-preserved and fresh SVF, our results are in agreement with what was reported by Costa et al. for fresh SVF. That study showed the spontaneous formation of a network of capillaries, with increased secretions of several angiogenic modulators from 5 to 8 days of culture. All of this, of course, occurred in the absence of extrinsic angiogenic factors [13]. Collagen scaffolds also provided structural and mechanical support and permitted significant capillary formation within its structure in vitro. The ability of collagen, as a three-dimensional matrix, to support the adherence and proliferation of endothelial cells in vitro has been observed for several years [48]. While mammalian collagen is the standard, new collagen sources have emerged. The skin and bones of several marine organisms are abundant in collagen, presenting very similar characteristics to that of mammalian origin. Blue shark skin collagen demonstrated a comparable chondrogenic differentiation of human adipose stem cells compared to the commercial alternative comprising bovine collagen [27]. In the case of a direct comparison between collagen from marine tilapia skin and bovine skin collagen, both showed a similar performance in a wound-healing scenario, allowing fibroblasts infiltration, vascularization, reduced inflammation, and collagen deposition [49]. In fact, the existing differences are only noticeable in proline and hydroxyproline contents [27]. The lower content of this amino acids in comparison with mammalian collagen, affects the denaturation temperature and, consequently, its thermal stability, resulting in a faster degradation of scaffolds [50]. Despite presenting a faster degradation rate due to its lower denaturation temperature, the use of crosslinkers allowed the stabilization of sponges. The large pores created (averaging 250–300 µm) [27] with highly interconnective microporosities positively influenced endothelial cell migration, rearrangement, and vessel density. Microporosity is considered critical for the new vessel’s size and number [51,52]. High pore interconnectivity results in significantly higher blood vessel density in vitro and increased blood vessel density and average invasion depth after implantation [53]. These architectural features present in the sponges used in this study allowed for the cell migration, proliferation, and organization of vascular networks, validating marine-derived collagen as a suitable raw material for TE of vascularized tissues. This alternative source of collagen for TE products not only surpasses disease-transmission concerns, such as bovine spongiform encephalopathy (BSE) [22], but also religious constraints, making it suitable for broader applications. Furthermore, new applications of marine by-products are of extreme importance, especially those that are environmentally friendly [54]. Its isolation represents low costs, creating value from products that are considered wasteful for the fish transformation industry.

To assess the in ovo functionality of the created prevascular network, a CAM assay was performed. The membrane of chick embryo provides a non-innervated and rapidly growing vascular bed, which can serve as a blood supply for engineered tissues and, therefore, be a useful model for testing prevascularization strategies [55]. In particular, its use has been reported in approaches ranging from cell sheets [56] to spheroids [57]. Herein, prevascularized collagen sponges were implanted onto the CAM and collected after 4 days. Empty sponges were implanted as controls. Both prevascularized and empty sponges were able to recruit blood vessels from the host in the implant region. Nonetheless, this effect was significantly improved for prevascularized constructs, suggesting that the presence of SVF can accelerate graft’s vascularization in ovo. Furthermore, the rapid infiltration of the implanted construct by host cells, without visible inflammatory response, revealed a superior integration with CAM when SVF cells were present. It is known that the cell complexity present in SVF can have an effect on neovascularization in ischemic tissues [58,59]. By injecting SVF in a hind limb ischemic mouse model, previous studies demonstrated that SVF cells not only improved blood flow [58,59] but were also able to integrate blood vessel linings [58]. It is thought that this improvement is mainly mediated by angiogenic cytokines secreted from implanted cells [60]. Since the latter two studies were conducted by injecting cells, the residence time for implanted cells at the injured site was probably reduced; thus, the release of angiogenic stimulators is limited in time. While the encapsulation of SVF can improve residency times, the creation of a 3D prevascular network prior to implantation [61] may further accelerate the vascularization of the graft in vivo through a rapid connection to recipient blood vessels [61,62]. In fact, the maturation of the created prevascular network also influences the angiogenic potential. Cerino et al. demonstrated that faster inosculation and the enhanced survival of transplanted cells in full-thickness rat wounds is associated to pericytes, for which its number was significantly higher in more mature constructs [62]. In the present study, it was clearly demonstrated that implanted human cells, namely CD31-positive cells, were able to integrate newly formed blood vessels at the interface between the CAM and implanted sponges. While this was not specifically tested, no evidence of prevascular network inosculation with the host’s circulation was found. Nevertheless, ISH and CD31 results, together with the demonstrated increase in vessel recruitment from the host, show that cryopreserved SVF had a positive effect on vascularization after implantation, underscoring the potential of this fraction for TE applications. 

## 4. Materials and Methods

### 4.1. Collagen Acid Extraction 

Collagen was extracted from blue shark (Prionace glauca) skin at Instituto de Investigaciones Marinas (CSCI, Vigo, Spain), according to a previously described protocol [27]. Blue shark skin was stirred with 0.1M NaOH in a cold room (3–5 °C) for 24 h to remove non-collagenous proteins and pigments. After a centrifugation step, the remaining pellet was washed with distilled water and incubated overnight with 0.5 M acetic acid under agitation to start the acid extraction process. The obtained extract was centrifuged at 10 °C, and the supernatant dialyzed against distilled water for 2 days in a cold room (3–5 °C). Finally, the obtained collagen extract was freeze-dried.

### 4.2. Collagen Sponge Fabrication

The production of highly interconnective microporous collagen sponges was performed as previously described [27]. Briefly, the previously extracted collagen was solubilized at 1% (*w/v*) concentration in a 10 mM hydrochloric acid (HCl, Sigma-Aldrich, St. Louis, MO, USA) solution. Cryogelation and crosslinking reactions were carried out at −20 °C for 4 h by the addition of 1-[3-(dimethylamino) propyl]-3-ethylcarbodiimide hydrochloride (EDC) (Mw: 191,70 g.mol^−1^, Sigma-Aldrich, St. Louis, MO, USA) at 60 mM of final concentration. To remove the residual crosslinker, cryogels were rinsed with distilled water before freeze drying.

### 4.3. Isolation and Cryopreservation of Human Adipose-Derived SVF Cells

Human subcutaneous adipose tissues were obtained from surgical procedures performed at Hospital de S. João (Porto), after obtaining the patient’s written informed consent, and within the scope of a collaboration protocol approved by the ethical committees of both institutions for this work (Comissão de Ética do Hospital de S. João/University of Minho: 217/19; CEICVS 008/2019). SVF was obtained as previously described [13]. Briefly, adipose tissue was digested with a collagenase type II (Sigma Aldrich, St. Louis, MO, USA) solution of 0.05% (*w/v*), for 45 min at 37 °C under agitation. After centrifugation, the obtained SVF was incubated with red blood lysis buffer and centrifuged, and the supernatant was resuspended in Minimum Essential Medium alpha-modification (α-MEM) (Life Technologies, Carlsbad, CA, USA) supplemented with 10% fetal bovine serum (FBS) and 1% antibiotic/antimycotic. Cell nuclei were stained using a solution of 3% (*v/v*) acetic acid (VWR, Lutterworth, UK) and 0.05 wt % methylene blue (Sigma Aldrich, St. Louis, MO, USA) in water to count nucleated cells. Finally, cells were cryopreserved in 10% (*v/v*) dimethyl sulfoxide (DMSO) in FBS with a controlled freeze rate of 1 °C/min for at least 7 days.

### 4.4. Cell Seeding

After thawing, a pool of five different donors was made. SVF was seeded on collagen sponges by dispensing 25 µL on top of the dried sponges and another 25 µL at the bottom of a 50 μL cell suspension comprising 1.5 × 10^6^ cells. Constructs were incubated for 1 h at 37 °C, 5% CO_2_, to allow maximum cell entrapment within the structures, and then fresh medium was added to a total volume of 1 mL. Constructs were cultured for 7 days in α-MEM to allow the formation of capillary-like structures.

### 4.5. Immunocytochemistry

After in vitro culture, constructs were fixed in a 10% (*v/v*) buffered formalin solution. A 3% (*w/v*) BSA was used to block non-specific binding, and cells were incubated overnight at 4 °C with primary antibody mouse anti-human CD31 (M0823) (1:30) (Dako, Cambridge, UK) and rabbit anti-human CD146 (ABCAAB75769) (1:50) (VWR, Lutterworth, UK). After repeated washes in PBS, secondary antibody Alexa Fluor 594 donkey anti-mouse (1:500) and AlexaFluor 488 donkey anti-rabbit (Molecular probes, Eugene, OR, USA) were incubated with cells for 4 h at room temperature. After washing with PBS, cell nuclei were counterstained with DAPI. The presence of capillary-like structures was verified in a confocal laser scanning microscope (SP8 Leica Microsystems CMS GmbH, Wetzlar, Germany).

### 4.6. Secretome Analysis

In order to analyze the expression profile of angiogenesis-related proteins during the prevascularization process, conditioned media were collected and centrifuged to remove cell debris after 5 and 7 days of culture. A control with basal media was also set up. Secreted proteins were analyzed using a Proteome Profiler human angiogenesis array (R&D Systems; Minneapolis, MN, USA) in accordance with manufacturer guidelines. Briefly, conditioned media were incubated with an assay-specific detection antibody cocktail for 1 h at room temperature. After a membrane blocking step, samples containing antibody cocktail were added to the respective membrane and incubated overnight at 4 °C under shaking. Membranes were then washed with 1× wash buffer, incubated with streptavidin-HRP for 30 min, and washed again. Membranes were incubated with Chemi Reagent Mix for 1 min, and spots detected by using chemiluminescence in an Odyssey Fc Imaging System (LI-COR, Lincoln, NE, USA) and densitometry were quantified using Image studio 5.2 software (LI-COR, Lincoln, NE, USA)

### 4.7. In ovo Implantation

A CAM assay was performed as previously described [57,63]. White fertilized chicken eggs were incubated at 37 °C in a temperature incubator (Termaks KB8000, Bergen, Norway) for 3 days. After this, a window was opened into the shell to evaluate embryo viability. Prevascularized sponges (*n* = 12) were implanted on the CAM at day 10 of embryonic development, and the eggs returned to the incubator at 37 °C. Control groups with empty materials (*n* = 12) and without the material (*n* = 12) were also set up. After 4 days of implantation, embryos were sacrificed with 4 % (*v/v*) paraformaldehyde and subsequent incubation at −80 °C for 10 min. Then, the implanted materials with adjacent portions of CAM were cut and fixed with 4% (*v/v*) paraformaldehyde. Ex ovo images were captured using a Stemi 2000-C stereo microscope (Zeiss, Oberkochen, Germany).

### 4.8. New Vessel Quantification

The obtained ex ovo images were processed using ImageJ 1.52a (National Institutes of Health, Bethesda, MD, USA). Images were cropped to a defined area of 500 × 500 pixels, considering the implanted construct in the center of the image. The number of new/recruited vessels growing radially towards the constructed area was quantified by manual counting, in a blind fashion, by three independent operators.

### 4.9. Histological Analysis

After formalin fixation, CAM explants were processed in a MICRON STP120-2 spin tissue processor (MICRON, Walldorf, Germany), embedded in paraffin (Thermo Scientific, Waltham, MA, USA), and serially sectioned into 4 µm-thick sections.

### 4.10. Hematoxylin and Eosin Staining

Hematoxylin and eosin (H&E) staining was performed in CAM sections. Briefly, sections were deparaffinized with xylene, rehydrated in graded ethanol series and stained with hematoxylin and eosin in an MICROM HMS740 automatic stainer (MICROM, Walldorf, Germany). Afterwards, sections were dehydrated and mounted with resinous mounting medium Entellan^®^ (Merck, Darmstadt, Germany). Histological sections were analyzed under a Leica DM750 microscope (Leica, Wetzlar, Germany).

### 4.11. Immunohistochemistry

CAM sections were deparaffinized and rehydrated in an MICROM HMS740 automatic stainer (MICROM, Walldorf, Germany). Immunohistochemical analysis was performed using a streptavidin–biotin peroxidase complex system. Briefly, after rehydration, slides were subjected to heat-induced antigen-retrieval with 10 mM citrate buffer at pH = 6 for 2 min at 98 °C. The slides were washed with PBS and then incubated with 3% hydrogen peroxide for 10 min to inactivate endogenous peroxidases. Another washing step was performed, and nonspecific binding was blocked with a 2.5% (*v/v*) horse serum (Vector Labs, Newark, CA, USA) for 30 min, before overnight incubation at 4 °C with mouse anti-human CD31 (1:30) (Dako, Cambridge, UK). Sections were washed with 0.1% tween in PBS and incubated with a secondary biotinylated antibody (Vector Labs, Newark, CA, USA) for 20 min. After thoroughly washed with 0.1% tween in PBS, samples were incubated with streptavidin-HRP (Vector Labs, Newark, CA, USA) for 20 min, followed by 3,3′diaminobenzidine (DAB) incubation (Vector Labs, Newark, CA, USA). Finally, all sections were counterstained with Mayer’s hematoxylin, dehydrated and mounted with resinous mounting medium Entellan® (Merck, Darmstadt, Germany).

### 4.12. In Situ Hybridization 

The presence of human cells within the implantation area was assessed using a human-specific DNA probe, according to the respective detection system BIO-AP REMBRANDT® Universal DISH & Detection kit (PanPath, Budel, The Netherlands). Briefly, after deparaffinization, proteolytic digestion was performed using a pepsin-HCL solution for 30 min at 37 °C, followed by dehydration in graded ethanol series. Sections were air-dried, and 1 drop of the probe was applied and covered with a coverslip. Samples were incubated at 95 °C for 5 min for DNA denaturation and then for 16 h at 37 °C in a moisturized environment for hybridization to occur. Samples were then washed in Tris-buffered saline (TBS) and incubated for 10 min with the stringency wash buffer. After rinsing with TBS, the detection was performed, and color was permitted to develop for 5 min at 37 °C in the dark. Samples were washed with water, counterstained with nuclear fast red, and observed under a Leica DM750 microscope (Leica, Wetzlar, Germany).

## 5. Conclusions

This study demonstrates that cryopreservation did not affect the capacity of SVF to spontaneously create an in vitro prevascular network in the absence of angiogenic growth factor supplementation in 3D conditions. Moreover, the angiogenic potential of SVF was further demonstrated after the in ovo implantation of prevascularized sponges resulting in improved vessel recruitment and improved construct integration within the CAM tissue. All together, these results demonstrate that the use of cryopreserved SVF, combined with marine-derived collagen, allows a simplified and cost-efficient method for assistance in the vascularization of TE constructs.

## Data Availability

Not applicable.

## References

[1] Rouwkema J., Koopman B.F., van Blitterswijk C., Dhert W., Malda J. (2009). Supply of Nutrients to Cells in Engineered Tissues. Biotechnol. Genet. Eng. Rev..

[2] Clark E.R., Clark E.L. (1939). Microscopic observations on the growth of blood capillaries in the living mammal. Am. J. Anat..

[3] Nishida K., Yamato M., Hayashida Y., Watanabe K., Yamamoto K., Adachi E., Nagai S., Kikuchi A., Maeda N., Watanabe H. (2004). Corneal Reconstruction with Tissue-Engineered Cell Sheets Composed of Autologous Oral Mucosal Epithelium. N. Engl. J. Med..

[4] Miyagawa S., Sawa Y., Sakakida S., Taketani S., Kondoh H., Memon I.A., Imanishi Y., Shimizu T., Okano T., Matsuda H. (2005). Tissue Cardiomyoplasty Using Bioengineered Contractile Cardiomyocyte Sheets to Repair Damaged Myocardium: Their Integration with Recipient Myocardium. Transplantation.

[5] Shafiee S., Shariatzadeh S., Zafari A., Majd A., Niknejad H. (2021). Recent Advances on Cell-Based Co-Culture Strategies for Prevascularization in Tissue Engineering. Front. Bioeng. Biotechnol..

[6] Kniebs C., Kreimendahl F., Köpf M., Fischer H., Jockenhoevel S., Thiebes A.L. (2020). Influence of Different Cell Types and Sources on Pre-Vascularisation in Fibrin and Agarose–Collagen Gels. Organogenesis.

[7] Qian Z., Sharma D., Jia W., Radke D., Kamp T., Zhao F. (2019). Engineering stem cell cardiac patch with microvascular features representative of native myocardium. Theranostics.

[8] Jackson C., Nguyen M. (1997). Human microvascular endothelial cells differ from macrovascular endothelial cells in their expression of matrix metalloproteinases. Int. J. Biochem. Cell Biol..

[9] Sacharidou A., Stratman A.N., Davis G.E. (2012). Molecular Mechanisms Controlling Vascular Lumen Formation in Three-Dimensional Extracellular Matrices. Cells Tissues Organs.

[10] Sun Y., Chen S., Zhang X., Pei M. (2019). Significance of Cellular Cross-Talk in Stromal Vascular Fraction of Adipose Tissue in Neovascularization. Arter. Thromb. Vasc. Biol..

[11] Bourin P., Bunnell B.A., Casteilla L., Dominici M., Katz A.J., March K.L., Redl H., Rubin J.P., Yoshimura K., Gimble J.M. (2013). Stromal cells from the adipose tissue-derived stromal vascular fraction and culture expanded adipose tissue-derived stromal/stem cells: A joint statement of the International Federation for Adipose Therapeutics and Science (IFATS) and the International Society for Cellular Therapy (ISCT). Cytotherapy.

[12] Ramakrishnan V.M., Boyd N.L. (2018). The Adipose Stromal Vascular Fraction as a Complex Cellular Source for Tissue Engineering Applications. Tissue Eng. Part B Rev..

[13] Costa M., Cerqueira M.T., Santos T.C., Sampaio-Marques B., Ludovico P., Marques A.P., Pirraco R.P., Reis R.L. (2017). Cell sheet engineering using the stromal vascular fraction of adipose tissue as a vascularization strategy. Acta Biomater..

[14] Mytsyk M., Cerino G., Reid G., Sole L., Eckstein F., Santer D., Marsano A. (2021). Long-Term Severe In Vitro Hypoxia Exposure Enhances the Vascularization Potential of Human Adipose Tissue-Derived Stromal Vascular Fraction Cell Engineered Tissues. Int. J. Mol. Sci..

[15] Coppola D., Oliviero M., Vitale G.A., Lauritano C., D’Ambra I., Iannace S., De Pascale D. (2020). Marine Collagen from Alternative and Sustainable Sources: Extraction, Processing and Applications. Mar. Drugs.

[16] Heino J. (2007). The collagen family members as cell adhesion proteins. BioEssays.

[17] Marino D., Luginbühl J., Scola S., Meuli M., Reichmann E. (2014). Bioengineering Dermo-Epidermal Skin Grafts with Blood and Lymphatic Capillaries. Sci. Transl. Med..

[18] Nilforoushzadeh M.A., Sisakht M.M., Amirkhani M.A., Seifalian A.M., Banafshe H.R., Verdi J., Nouradini M. (2020). Engineered skin graft with stromal vascular fraction cells encapsulated in fibrin–collagen hydrogel: A clinical study for diabetic wound healing. J. Tissue Eng. Regen. Med..

[19] Lugo-Cintrón K.M., Ayuso J.M., White B.R., Harari P.M., Ponik S.M., Beebe D.J., Gong M.M., Virumbrales-Muñoz M. (2020). Matrix density drives 3D organotypic lymphatic vessel activation in a microfluidic model of the breast tumor microenvironment. Lab Chip.

[20] McCoy M.G., Seo B.R., Choi S., Fischbach C. (2016). Collagen I hydrogel microstructure and composition conjointly regulate vascular network formation. Acta Biomater..

[21] Montaño I., Schiestl C., Schneider J., Pontiggia L., Luginbühl J., Biedermann T., Böttcher-Haberzeth S., Braziulis E., Meuli M., Reichmann E. (2010). Formation of Human Capillaries In Vitro: The Engineering of Prevascularized Matrices. Tissue Eng. Part A.

[22] European Medicines Agency (2011). Note for guidance on minimising the risk of transmitting animal spongiform encephalopathy agents via human and veterinary medicinal products (EMA/410/01 rev.3). Off. J. Eur. Union.

[23] Sotelo C.G., Comesaña M.B., Ariza P.R., Pérez-Martín R.I. (2015). Characterization of Collagen from Different Discarded Fish Species of the West Coast of the Iberian Peninsula. J. Aquat. Food Prod. Technol..

[24] Fowler G.M., Campana S.E. (2009). Commercial by-catch rates of blue shark (prionace glauca) from longline fisheries in the Canadian Atlantic. Collect. Vol. Sci. Pap. ICCAT.

[25] Elango J., Lee J.W., Wang S., Henrotin Y., De Val J.E.M.S., Regenstein J.M., Lim S.Y., Bao B., Wu W. (2018). Evaluation of Differentiated Bone Cells Proliferation by Blue Shark Skin Collagen via Biochemical for Bone Tissue Engineering. Mar. Drugs.

[26] Nomura Y., Yamano M., Hayakawa C., Ishii Y., Shirai K. (1997). Structural Property and in Vitro Self-assembly of Shark Type I Collagen. Biosci. Biotechnol. Biochem..

[27] Diogo G.S., Carneiro F., Freitas-Ribeiro S., Sotelo C.G., Pérez-Martín R.I., Pirraco R.P., Reis R.L., Silva T.H. (2020). Prionace glauca skin collagen bioengineered constructs as a promising approach to trigger cartilage regeneration. Mater. Sci. Eng. C.

[28] Joshi V.S., Lei N.Y., Walthers C.M., Wu B., Dunn J.C. (2013). Macroporosity enhances vascularization of electrospun scaffolds. J. Surg. Res..

[29] Perets A., Baruch Y., Weisbuch F., Shoshany G., Neufeld G., Cohen S. (2003). Enhancing the vascularization of three-dimensional porous alginate scaffolds by incorporating controlled release basic fibroblast growth factor microspheres. J. Biomed. Mater. Res..

[30] Oliviero O., Ventre M., Netti P. (2012). Functional porous hydrogels to study angiogenesis under the effect of controlled release of vascular endothelial growth factor. Acta Biomater..

[31] Sharma D., Ross D., Wang G., Jia W., Kirkpatrick S.J., Zhao F. (2019). Upgrading prevascularization in tissue engineering: A review of strategies for promoting highly organized microvascular network formation. Acta Biomater..

[32] Liao H., He H., Chen Y., Zeng F., Huang J., Wu L., Chen Y. (2014). Effects of long-term serial cell passaging on cell spreading, migration, and cell-surface ultrastructures of cultured vascular endothelial cells. Cytotechnology.

[33] Wittmann K., Dietl S., Ludwig N., Berberich O., Hoefner C., Storck K., Blunk T., Bauer-Kreisel P. (2015). Engineering Vascularized Adipose Tissue Using the Stromal-Vascular Fraction and Fibrin Hydrogels. Tissue Eng. Part A.

[34] Collen D. (1999). The Plasminogen (Fibrinolytic) System. Thromb. Haemost..

[35] Pepper M.S., Sappino A.P., Montesano R., Orci L., Vassalli J.-D. (1992). Plasminogen activator inhibitor-1 is induced in migrating endothelial cells. J. Cell. Physiol..

[36] Pepper M., Montesano R. (1990). Proteolytic balance and capillary morphogenesis. Cell Differ. Dev..

[37] Kraling B., Wiederschain D., Boehm T., Rehn M., Mulliken J., Moses M. (1999). The role of matrix metalloproteinase activity in the maturation of human capillary endothelial cells in vitro. J. Cell Sci..

[38] Charo I.F., Taubman M.B. (2004). Chemokines in the Pathogenesis of Vascular Disease. Circ. Res..

[39] Jaffe E., Ruggiero J., Falcone D. (1985). Monocytes and macrophages synthesize and secrete thrombospondin. Blood.

[40] Koch A.E., Polverini P.J., Kunkel S.L., Harlow L.A., DiPietro L.A., Elner V.M., Elner S.G., Strieter R.M. (1992). Interleukin-8 as a macrophage-derived mediator of angiogenesis. Science.

[41] Li A., Dubey S., Varney M.L., Dave B.J., Singh R.K. (2003). IL-8 Directly Enhanced Endothelial Cell Survival, Proliferation, and Matrix Metalloproteinases Production and Regulated Angiogenesis. J. Immunol..

[42] Hong K.H., Ryu J., Han K.H. (2005). Monocyte chemoattractant protein-1–induced angiogenesis is mediated by vascular endothelial growth factor-A. Blood.

[43] Yamada M., Kim S., Egashira K., Takeya M., Ikeda T., Mimura O., Iwao H. (2003). Molecular Mechanism and Role of Endothelial Monocyte Chemoattractant Protein-1 Induction by Vascular Endothelial Growth Factor. Arter. Thromb. Vasc. Biol..

[44] Ma J., Wang Q., Fei T., Han J.-D.J., Chen Y.-G. (2006). MCP-1 mediates TGF-β–induced angiogenesis by stimulating vascular smooth muscle cell migration. Blood.

[45] Mendes L.F.F., Pirraco R.P., Szymczyk W., Frias A.M., Santos T.C., Reis R.L., Marques A.P. (2012). Perivascular-Like Cells Contribute to the Stability of the Vascular Network of Osteogenic Tissue Formed from Cell Sheet-Based Constructs. PLoS ONE.

[46] McIlroy M., O’Rourke M., McKeown S.R., Hirst D.G., Robson T. (2006). Pericytes influence endothelial cell growth characteristics: Role of plasminogen activator inhibitor type 1 (PAI-1). Cardiovasc. Res..

[47] Lobov I.B., Brooks P.C., Lang R.A. (2002). Angiopoietin-2 displays VEGF-dependent modulation of capillary structure and endothelial cell survival in vivo. Proc. Natl. Acad. Sci. USA.

[48] Montesano R., Orci L., Vassalli P. (1983). In vitro rapid organization of endothelial cells into capillary-like networks is promoted by collagen matrices. J. Cell Biol..

[49] Chen J., Gao K., Liu S., Wang S., Elango J., Bao B., Dong J., Liu N., Wu W. (2019). Fish Collagen Surgical Compress Repairing Characteristics on Wound Healing Process In Vivo. Mar. Drugs.

[50] Gauza-Włodarczyk M., Kubisz L., Mielcarek S., Włodarczyk D. (2017). Comparison of thermal properties of fish collagen and bovine collagen in the temperature range 298–670 K. Mater. Sci. Eng. C.

[51] Bai F., Wang Z., Lu J., Liu J., Chen G., Lv R., Wang J., Lin K., Zhang J., Huang X. (2010). The Correlation Between the Internal Structure and Vascularization of Controllable Porous Bioceramic Materials In Vivo: A Quantitative Study. Tissue Eng. Part A.

[52] Somo S.I., Akar B., Bayrak E.S., Larson J.C., Appel A.A., Mehdizadeh H., Cinar A., Brey E.M. (2015). Pore Interconnectivity Influences Growth Factor-Mediated Vascularization in Sphere-Templated Hydrogels. Tissue Eng. Part C Methods.

[53] Mehdizadeh H., Sumo S., Bayrak E.S., Brey E.M., Cinar A. (2013). Three-dimensional modeling of angiogenesis in porous biomaterial scaffolds. Biomaterials.

[54] Kirkfeldt T.S., Santos C.F. (2021). A Review of Sustainability Concepts in Marine Spatial Planning and the Potential to Supporting the UN Sustainable Development Goal 14. Front. Mar. Sci..

[55] Moreno-Jiménez I., Kanczler J.M., Hulsart-Billstrom G., Inglis S., Oreffo R.O. (2017). The Chorioallantoic Membrane Assay for Biomaterial Testing in Tissue Engineering: A Short-Term In Vivo Preclinical Model. Tissue Eng. Part C Methods.

[56] Silva A.S., Santos L.F., Mendes M.C., Mano J.F. (2020). Multi-layer pre-vascularized magnetic cell sheets for bone regeneration. Biomaterials.

[57] Feijão T., Neves M.I., Sousa A., Torres A.L., Bidarra S.J., Orge I.D., Carvalho D.T., Barrias C.C. (2021). Engineering injectable vascularized tissues from the bottom-up: Dynamics of in-gel extra-spheroid dermal tissue assembly. Biomaterials.

[58] Sumi M., Sata M., Toya N., Yanaga K., Ohki T., Nagai R. (2007). Transplantation of adipose stromal cells, but not mature adipocytes, augments ischemia-induced angiogenesis. Life Sci..

[59] Koh Y.J., Koh B.I., Kim H., Joo H.J., Jin H.K., Jeon J., Choi C., Lee D.H., Chung J.H., Cho C.-H. (2011). Stromal Vascular Fraction From Adipose Tissue Forms Profound Vascular Network Through the Dynamic Reassembly of Blood Endothelial Cells. Arter. Thromb. Vasc. Biol..

[60] Nakagami H., Maeda K., Morishita R., Iguchi S., Nishikawa T., Takami Y., Kikuchi Y., Saito Y., Tamai K., Ogihara T. (2005). Novel Autologous Cell Therapy in Ischemic Limb Disease Through Growth Factor Secretion by Cultured Adipose Tissue–Derived Stromal Cells. Arter. Thromb. Vasc. Biol..

[61] Klar A.S., Güven S., Zimoch J., Zapiórkowska N.A., Zapiórkowska T., Böttcher-Haberzeth S., Meuli-Simmen C., Martin I., Scherberich A., Reichmann E. (2016). Characterization of vasculogenic potential of human adipose-derived endothelial cells in a three-dimensional vascularized skin substitute. Pediatr. Surg. Int..

[62] Cerino G., Gaudiello E., Muraro M.G., Eckstein F., Martin I., Scherberich A., Marsano A. (2017). Engineering of an angiogenic niche by perfusion culture of adipose-derived stromal vascular fraction cells. Sci. Rep..

[63] Oliveira C., Granja S., Neves N., Reis R.L., Baltazar F., Silva T.H., Martins A. (2019). Fucoidan from Fucus vesiculosus inhibits new blood vessel formation and breast tumor growth in vivo. Carbohydr. Polym..

